# Genetic and Environmental Factors Influencing the Production of Select Fungal Colorants: Challenges and Opportunities in Industrial Applications

**DOI:** 10.3390/jof9050585

**Published:** 2023-05-18

**Authors:** Lan Lin, Tong Zhang, Jianping Xu

**Affiliations:** 1 Key Laboratory of Developmental Genes and Human Diseases (MOE), School of Life Science and Technology, Southeast University, Nanjing 210096, China; linl04@seu.edu.cn; 2 Department of Bioengineering, Medical School, Southeast University, Nanjing 210009, China; 213181266@seu.edu.cn; 3 Department of Biology, McMaster University, Hamilton, ON L8S 4K1, Canada

**Keywords:** fungi, colorants, metabolic regulation, carotenoids, melanins, *Monascus* pigments (Mps)

## Abstract

Natural colorants, mostly of plant and fungal origins, offer advantages over chemically synthetic colorants in terms of alleviating environmental pollution and promoting human health. The market value of natural colorants has been increasing significantly across the globe. Due to the ease of artificially culturing most fungi in the laboratory and in industrial settings, fungi have emerged as the organisms of choice for producing many natural colorants. Indeed, there is a wide variety of colorful fungi and a diversity in the structure and bioactivity of fungal colorants. Such broad diversities have spurred significant research efforts in fungi to search for natural alternatives to synthetic colorants. Here, we review recent research on the genetic and environmental factors influencing the production of three major types of natural fungal colorants: carotenoids, melanins, and polyketide-derived colorants. We highlight how molecular genetic studies and environmental condition manipulations are helping to overcome some of the challenges associated with value-added and large-scale productions of these colorants. We finish by discussing potential future trends, including synthetic biology approaches, in the commercial production of fungal colorants.

## 1. Introduction

Fungi are ubiquitously distributed on our planet and play important roles in the health of plants, animals, humans, and the environment. Most fungi are able to produce various extracellular enzymes to decompose organic matter, as well as perform a diversity of metabolic functions involved in both anabolism and catabolism. These traits make fungi an excellent reservoir for producing enzymes, as well as organic acids and alcohols, at commercial scale [1]. In addition to producing those abovementioned bulk industrial goods, fungi can produce other secondary metabolites such as colorants, which have been widely used in the food, cosmetic, textile, and leather industries [2].

Globally, the negative effects of synthetic food colorants consumed by humans, including but not limited to hyperactivity in children, allergies, cancer, and reproductive disorders, are increasingly being reported and have propelled our exploration of natural alternatives [3,4]. From the global viewpoint, a sustainable economy calls for materials and processes that adversely affect the environment as little as possible. Natural resources, including both natural compounds and natural bioprocesses, are being investigated as alternatives to synthetic chemicals. For example, the utilization of yellow colorants tartrazine and sunset, as well as red coloring compounds such as carmine and amaranth, have become increasingly restricted in developed countries according to the regulations issued by the World Health Organization (WHO) [5]. Indeed, the declining acceptability of synthetic colorants is observed worldwide, predominantly attributed to their nonbiodegradability after being released into the ecosystem, as well as increasing links between the intake of artificial food colors and side-effects on human health [3,6].

In addition to their dyeing ability, fungal colorants have been found to possess a multitude of structures with diverse bioactivities such as antitumor, anti-obesity, cholesterol-lowering and/or anti-atherosclerotic, anti-Alzheimer’s disease, antioxidative, and immunosuppressive activities [6]. Such broad activities make them stand out as pharmacophores in the discovery and development of drugs to fight against miscellaneous complicated diseases including cancers in humans. For readers interested in this specific topic, please refer to other publications for the structural diversity and bioactivities of fungal colorants association with human health [2,7].

In contrast to the colorants derived from plants such as carotenoids, fungi, as a whole, represent an enormous arsenal of bio-colorants that are not subjected to seasonal/geographic variations or lengthy cultivation time. With developments in advanced cell culture techniques, highly automated fermentation devices (e.g., large bioreactors), and innovative downstream bioprocessing, fungi have increasingly become attractive host organisms for producing colorants for human consumptions. For industrial application of fungal colorants, it is essential to develop fungal strains that can produce abundant desirable colorants. In addition, our knowledge about the regulation of colorant biosynthetic pathways can be harnessed via metabolic engineering approaches. Metabolic engineering is the improvement of cellular properties through the modification of specific biochemical reactions, with the use of recombinant DNA technology [8]. These approaches may include increasing carbon fluxes for desired intermediates/end-products or lowering carbon fluxes to reduce unwanted metabolites. Biotechnological productions of fungal colorants are also influenced by a variety of other factors such as the types of fermenters, cultivation parameters, temperature, availability of carbon/nitrogen sources and other nutrients, and supply of oxygen.

Colorants include all colorful substances and are commonly called dyes and pigments across diverse industrial sectors. Although often used interchangeably, dyes are finer than pigments and unstable under UV, while pigments are typically stable under UV exposure. In addition, dyes are soluble in water and/or an organic solvent, while pigments are insoluble in both types of liquid media. Dyes are used to color substrates to which they have affinity. Pigments can be used to color any polymeric substrate but via a quite different mechanism from that of dyes, in that surface-only coloration is involved [9]. In this review, the term “colorant” is used to include both dyes and pigments, while the terms “pigment” and “dye” are used for specific categories of colorants.

This review summarizes recent research on the production and regulation of three major types of natural colorants produced by fungi: carotenoids, melanins, and polyketide-based colorants (i.e., *Monascus* pigments). These three major groups of fungal colorants were chosen here because they are economically among the most important and biologically among the best understood fungal colorants. They also exhibit a diversity of colors and have been used in a broad range of applications. They are classified on the basis of their chemical composition, biosynthetic pathways, and abundance in fungi. Fungal carotenoids are lipid-soluble colorants and contain an aliphatic polyene chain consisting of eight isoprene units, thereby exhibiting characteristic yellow, orange, or reddish colors. Quinones are oxidized derivatives of aromatic compounds, corresponding to a wide array of fungal colorants. Their coloration depends on their structure, such as naphthoquinones and anthraquinones. Fungal melanins are formed via a complex polymerization process involving quinones and free radicals; thus, the resultant compounds are often of brown or black hues [10]. Specifically, we reviewed the fungal colorant biosynthetic pathways, their intrinsic regulatory mechanisms, external factors that influence their production, and strategies to improve their yields. Further opportunities and trends in the industrial production of colorants via biotechnological approaches are also discussed.

## 2. Carotenoids

### 2.1. Carotenoids and Carotenoid Synthesis Pathways

**Figure 1:**
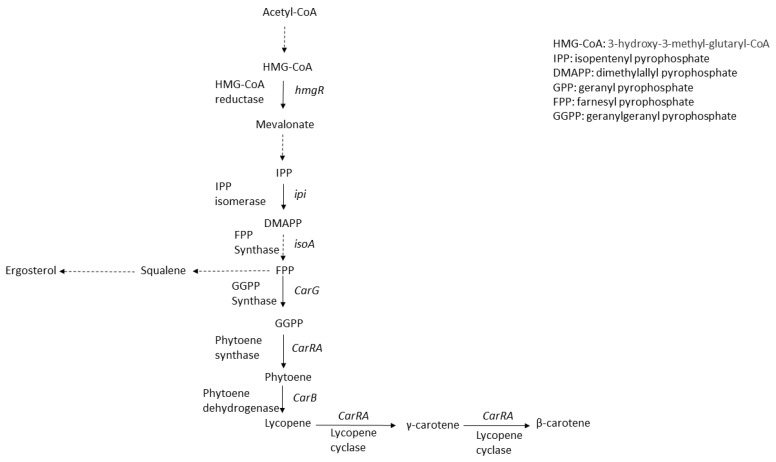
Biosynthetic pathway of β-carotene and branched pathway along with gene-coding enzymes involved in β-carotene metabolism of *Blakeslea trispora*. Dashed arrows indicate multiple-step catalytic conversion while black arrows indicate one-step conversion.

Carotenoids are lipophilic colorants with yellow, orange, and red hues. Via the isoprenoid (also known as terpenoid) pathway, these tetraterpenoid compounds are biosynthesized in most cellular organisms except animals. Fungal carotenoids are derived from 3-hydroxy-3-methyl-glutaryl-CoA (HMG-CoA) in the mevalonate (MVA) pathway, regulated by sophisticated genetic mechanisms, with the basic units being isopentenyl-pyrophosphate (IPP) and its isomer dimethylallyl-pyrophosphate (DMAPP) (Figure 1). The early biosynthetic reactions of carotenoids in fungi involve the sequential additions of IPP (isoprene, C5) units to generate geranyl pyrophosphate (GPP, C10), farnesyl pyrophosphate (FPP, C15), and geranylgeranyl pyrophosphate (GGPP, C20), followed by condensation of two molecules of GGPP to phytoene, and the successive desaturation of phytoene to yield lycopene [6]. The condensation step is catalyzed by a single enzyme, with its coding gene designated as *crtYB* (*Xanthophyllomyces dendrorhous*) or *carRA* (*Blakeslea trispora*), and the phytoene desaturation is catalyzed by the protein encoded by gene assigned as *crtI* (*X. dendrorhous*) or *carB (B. trispora)* depending on the fungal species [11]. Table 1 gives an overview on the key genes involved in the biosynthesis of carotenoids in fungi.

### 2.2. Production of Carotenoids

Amongst the carotenoid-producing microorganisms are bacteria, filamentous fungi, yeasts, and microalgae, such as *Blakeslea trispora*, *Phycomyces blakesleeanus*, *Flavobacterium* sp., *Phaffia* sp., and *Rhodotorula* sp. [17,18]. *Blakeslea trispora*, a member of the Mucorales order in fungi, has been described as a carotenoid hyperproducer, and it is widely used in industrial production of carotenoids worldwide.

In order to improve the β-carotene yield, genetic engineering approaches have been explored and harnessed, including heterologous expression of key biosynthetic genes by either plasmid-based transformation methods or chromosomal integration techniques. For example, attempts have been made using general chassis microorganisms including *E. coli*, *Saccharomyces cerevisiae*, and *Yarrowia lipolytica* as hosts to produce β-carotene.

In an early attempt to improve the carotenoid production, two key genes encoding isopentenyl pyrophosphate isomerase (IPI) and geranylgeranyl pyrophosphate synthase (GGPS) of β-carotene synthesis were cloned from *B. trispora*, the leading industrial producer for β-carotene, and their properties were functionally studied in the heterologous host *E. coli* [19]. The effect of exogenous GGPS activity on carotene biosynthesis was found to be dependent upon the elevation of IPP activity in *E. coli* with plasmid pACCAR16ΔcrtX. The introduction of GGPS and IPI genes boosted the production of β-carotene in *E. coli* from 0.5 to 0.95 mg/g dry weight. Results also facilitated the elucidation of the molecular mechanism of β-carotene biosynthesis and the provision of two novel genes for metabolic engineering of carotenoids [19]. Later on, in view of the drawbacks of plasmid-based heterologous expression systems such as instability and burden on growth of host cells, as well as decreases in biomass and product yield, genetic manipulation involving chromosome integration was implemented to enhance the carotene level. The mevalonate (MVA) pathway was divided into three modules, and two heterologous modules, namely, the *Hmg1*-*erg12* operon and the *mvaS-mvaA*-*mavD1* operon, were integrated into the *E. coli* chromosome. Subsequent to the chromosomal integration, modulation with promoters with varied strengths contributed to an increase of 26% in β-carotene yield without an adverse effect on biomass. Using a combinatory modulation of two key enzymes *mvaS* and *Hmg1* with a degenerate RBS (ribosome-binding site) library, β-carotene was reported to display a further elevated production by 51% [20].

Similarly, to achieve the goal of producing *β*-carotene in the heterologous non-carotenogenic host *S. cerevisiae*, related genes from carotenogenic yeast *Xanthophyllomyces dendrorhous*, namely, *crtE*, *crtI*, and *crtYB*, were studied [21]. These genes were known to encode a bifunctional phytoene synthase and lycopene cyclase in the biosynthesis of carotenoids in *X. dendrorhous*. Carotenogenic genes were successively integrated into the *S. cerevisiae* chromosome to enable the stable gene expression and, thus, production of carotenoids. To improve the carbon flux, isoprenoid genes encoding tHMG1 and GGPP synthase were introduced, which ultimately led to an elevated yield of carotenoid, reaching 5.9 mg/g dry weight in the resulting *S. cerevisiae* strain. Optimization of the gene expression associated with both isoprenoid and carotenoid biosynthetic pathways was proven crucial for the hyperproduction of *β*-carotene in *S. cerevisiae*.

As a generally recognized as safe (GRAS) microbe, the oleaginous yeast *Yarrowia lipolytica* possesses sufficient lipid bodies to store lipophilic compounds such as carotenoids. To enhance β-carotene production in this organism, a study was conducted by introducing the codon-optimized *CarRA* and *CarB* genes from *B. trispora* into *Y. lipolytica* into the chromosome to produce strain YL-C0 [22]. Subsequently, precise regulation of the metabolic balance of the β-carotene biosynthetic pathway in YL-C0 was achieved by minimizing the accumulation of intermediate metabolites. Remarkably, the β-carotene level was found to increase 21-fold to 12.5 mg/g dry biomass when minimizing HMG-CoA and FPP accumulation. The expression levels of the *CarRA* and *CarB* genes were further improved to reduce the accumulation of phytoene and lycopene. The overall yield of β-carotene was found to reach 1.7 g/L and 21.6 mg/g dry biomass. The above experimentation clearly demonstrated that the rate-limiting enzymes CarRA and CarB originated from *B. trispora* could display higher catalytic activity than the same enzymes from other microbes. Maintaining metabolic balance by minimizing the intermediate accumulation is a very effective strategy for elevating the level of β-carotene in the engineered heterologous hosts including chassis organisms, which would broaden the potential for large-scale production of carotenoids at industrial scale.

Mutagenesis work with the fungus *Mucor circinelloides* was recently performed for β-carotene overproduction using a combination of classical forward and modern reverse genetic techniques [23]. Two high-carotene-content mutants, namely, MU206 and MU218, among 26 mutants, were further investigated. Results revealed that the deletion in the above two mutants of the *crgA* gene, a well-known repressor of carotenoid biosynthesis, led to increased β-carotene accumulation. One strain derived from MU218 was capable of yielding up to 4 mg/g dry biomass of β-carotene [23].

In addition to genetic engineering approaches, optimization of culture conditions represents another approach to boost carotenoid production at the industrial scale. The parameters affecting carotenogenesis include light exposure, oxygen, temperature, and nitrogen and Ca^++^ availabilities [24]. For example, it has been reported that absence of Ca ions in the culture media would favor a higher carotenoid production than Ca^++^-containing media in the cases of *Mucor circinelloides* and *Amylomyces rouxii* [24].

Given that fungal carotenoid production is proposed as a natural mechanism to protect fungi against photo-oxidative damage in light-intensive habitats [25,26], it is not surprising that light would increase the production of carotenoids. Studies have shown that light-regulated carotenogenesis is common in filamentous fungi, particularly in the order of Mucorales. However, little is known about the mechanism of photoinduction of carotenoid accumulation and underlying mechanisms in *B. trispora*. Three photoreceptor genes of *B. trispora*, namely, BTWC-1A, BTWC-1B, and BTWC-1C, have been cloned, expressed, and subsequently characterized using bioinformatics approaches, followed by in vivo functional analyses of these genes constructed in *M. circinelloides* [13]. The mucorales *B. trispora* and *M. circinelloides* are closely related; many of their genes are highly conserved and have orthologous functions. The results enhanced our understanding of photo-regulated carotenoid production in *B. trispora* and provided promising prospects to achieve high yields of carotenoid in *B. trispora* by modulating the light-inducible carotenogenesis pathway. In a recent study, Ge et al. reported the first transcriptome work in *B. trispora* concerning global gene expression changes in response to blue light [27]. It was found that (1) the level of β-carotene increased threefold when transferred from darkness to blue-light exposure for 24 h, and (2) the upregulated transcriptional levels of *carRA* and *carB* were positively correlated with the elevated carotenoid yields. Further RNA-seq analysis revealed that 1124 genes were upregulated while 740 genes were downregulated in response to blue-light exposure. Among the blue-light-responsive genes, four previously identified genes as central to carotenoid biosynthesis pathways, namely, *carG1*, *carG3*, *carRA*, and *carB*, were prominently upregulated.

Similar to that reported in *B. trispora*, species in the genus of *Fusarium* such as *F. aquaeductuum* and *F. fujikuroi* also showed increased carotenoid production when transitioned from dark to light environments [28,29]. The light-stimulated carotenoid biosynthesis in *Fusarium fujikuroi* involved transcriptional regulation of the structural genes of the carotenoid biosynthetic pathway, *carRA*, *carB*, and *cart*. Additionally, the *carS* gene was identified as a regulatory gene for carotenoid production in *Fusarium.* Interestingly, mutation of *carS* resulted in transcriptional upregulation of the structural *car* genes and accumulation of a high level of carotenoids, consistent with its suppressive role [30,31]. The *F. fujikuroi* genome contains genes for different predicted photoreceptors, including the WC protein WcoA, the DASH cryptochrome CryD, and the Vivid-like flavoprotein VvdA. It was observed that the wildtype *F. fujikuroi* exhibited a biphasic response in light-induced kinetic experiments, with a sharp increase in carotenoid accumulation in the initial hours, followed by a transient arrest and a subsequent slower increase [32]. These results suggest that *F. fujikuroi* has a highly sophisticated regulatory system of carotenoid biosynthesis in response to illumination.

In addition to light exposure, the effect of temperature on β-carotene production was investigated in the recombinant strain of *S. cerevisiae* transformed with *crtE*, *crtYB*, and *crtI*, three carotenogenic genes from *Phaffia rhodozyma* (viz. *Xanthophyllomyces dendrorhous*) [33]. The recombinant *S*. *cerevisiae* cells could accumulate 258.8 μg/g dry weight of β-carotene at 20 °C, approximately 59-fold higher than those grown at 30 °C. The further introduction and expression of the catalytic domain of 3-hydroxy-3 methylglutaryl CoA (HMG-CoA) reductase in *S. cerevisiae* was shown to increase β-carotene contents to 528.8 μg/g dry weight at 20 °C, which was 27-fold higher than cells grown at 30 °C.

Of note, recent studies relevant to the topic of fermentation optimization have been highlighted in several studies, as exemplified by Kalra et al. (2020) [34] and Romano et al. (2018) [35], in which the “one strain many compounds (OSMAC)” strategy was proposed as a rational framework to elicit the production and discovery of new colorants by altering the cultivation parameters.

In addition to genetic engineering and culture optimization strategies, metabolic regulation has attracted academic and industrial interests to enhance carotenoid yield. Since the isoprenoid pathway provides the common precursors for the biosynthesis of carotenoid and related compounds, increasing the carbon flux through this pathway is an important strategy to achieve desirable rate, titer, and yield for lycopene and β-carotene.

Precursor supplementation could also be an effective means to improve carotenoid yield in certain chassis organisms such as *E. coli*. When recombinant *E. coli* was given 16.5 mM mevalonate and 2.5% (*w*/*v*) glycerol, β-carotene yield of 503 mg/L in terms of concentration and 49.3 mg/g in terms of dry biomass in content was achieved at 144 h in a flask culture, which was the highest level of carotenoid production in *E. coli* ever documented in the literature [36]. This was achieved using the foreign mevalonate pathway. Similarly, studies with recombinant *E. coli* by Alper et al. demonstrated that the rate of lycopene production was elevated from 11 mg/g DCW in 48 h to 18 mg/g DCW in 24 h by optimizing glucose feeding in a fermenter during fed-batch culture [37]. Considering that fermentative conditions in the bioreactor excel in the control of pH, dissolved oxygen, and substrate concentration in contrast to those in the flask culture, a short cultivation time (less than 144 h) would be expected after optimization of fermentative parameters using fed-batch culture. Fed-batch culture has the following advantages: (1) catabolite repression and Crabtree effects can be managed by limiting the substrate concentration; (2) high cell density can be obtained; (3) production of non-growth-related metabolites, i.e., secondary metabolites, can be increased. Indeed, previous work reported enhanced astaxanthin production in *Phaffia rhodozyma* via fed-batch culture with glucose and ethanol feeding [38].

The supplementation of chemical inhibitors or activators has been used to block or boost specific metabolic steps during carotenoid biosynthesis to attain the targeted colorants [39,40]. A decade ago, the inhibitor piperidine was found to be able to repress the carotenoid pathway at the lycopene step, in which piperidine at 500 ppm gave a 7.76-fold improvement of lycopene by inhibiting lycopene cyclase and, thus, blocking the ensuing conversion from lycopene to γ-carotene [40]. Another paradigm of metabolic regulation is documented to suppress the shunt pathways that compete for common precursors, as evidenced by the use of cerulenin and ketoconazole, in *M. circinelloides*, to inhibit fatty acid and sterol synthesis, which compete against carotenogenesis for acetyl-CoA and farnesyl diphosphate, respectively [40].

More recently, an investigation with *B*. *trispora* showed that SR5AL (steroid 5α-reductase-like) is a key gene associated with the response to trisporic acids and involved in regulating lycopene biosynthesis [41]. Trisporic acids, serving as sex hormones in members of the order Mucorales including *B. trispora*, are known to facilitate metabolic flux toward carotenoid biosynthesis although their effects are indirect. The abovementioned studies identified the SR5AL gene as a key regulatory gene for lycopene biosynthesis in *B. trispora* (−) in response to trisporic acid. Thus, the overexpression of the SR5AL gene might result in enhanced lycopene biosynthesis regardless of trisporic acid supplementation, thereby paving the road to increase the lycopene yields using single fermentation of *B. trispora* (−). It is widely recognized that *B. trispora* (−) mating type is the major lycopene producer, and the role of mating type (+) in lycopene synthesis may be replaced by genes involved in the synthesis of trisporic acid to enhance our ability for genetic manipulations to derive high-yield strains [41].

## 3. Melanins

### 3.1. Melanins and Melanin Synthesis Pathways

Melanins are usually found as fungal structural components, commonly presented in dark hues [42,43,44]. Most ascomycetes synthetize melanin through the polyketide pathway, using 1,8-dihydroxynaphthalene (1,8-DHN) as a precursor, which is designated the DHN–melanin pathway. In contrast, Basidiomycetes and some Ascomycetes adopt the DOPA–melanin pathways, using L-3,4-dihyroxyphenylalanine (L-DOPA) as a precursor in a route analogous to mammalian melanin biosynthesis [43]. Another route is pyomelanin produced via tyrosine degradation by *Aspergillus fumigatus* and *Penicillium chrysogenum* [45,46].

As an indispensable constituent of the cell wall for many fungi, melanin is known to protect organisms from environmental stresses. In several human pathogenic fungi, such as *Aspergillus fumigatus* and *Cryptococcus neoformans*, melanin was demonstrated to contribute to virulence. In the case of *A. fumigatus*, DHN–melanin is responsible for the pigmentation of conidia, the biosynthesis of which involves six genes [47]. Among these genes, *pksP*, the gene coding for polyketide synthase, was found to be a core element. It has been documented that DevR and another MADS box transcription factor, RlmA, could bind to the *pksP* promoter to induce the *pksP* expression at transcription level, thereby regulating melanin production [48].

To facilitate our understanding of how genetic manipulations have helped in enhancing melanin production in fungi, the genes associated with melanin biosynthesis in representative fungi are summarized in Table 2.

Due to their high heterogeneity, insolubility in organic solvents, hydrophobicity, and resistance to chemical degradation, appropriate extraction and purification methods are required for the application of melanins. Advances in the extraction procedures to obtain fungal melanins are described and discussed elsewhere [42,43,44]. Here, we focus on reviewing studies targeting the increased production and application of fungal melanins.

### 3.2. Production of Fungal Melanins

The search for novel fungal species capable of secreting large quantities of extracellular melanin has intensified in the field of melanin production. Success of this strategy may facilitate convenient and rapid downstream processing, while alleviating the environmental burden due to the reduction in contaminated effluents. In recent years, such a strategy has been employed by two independent groups. One group generated extracellular melanin in liquid culture of the isolated fungal species, as exemplified by *Amorphotheca resinae* and *Armillaria cepistipes*, with yields of 4.5 g/L within 14 days and 27.98 g/L within 161 days of cultivation, respectively [52]. An earlier investigation with a saprophytic fungus *Lachnum* YM 404, showed that extracellular melanins (LEM404) were extracted from its fermentation broth with a yield of 3.45 g/L [53]. The other group used mushrooms *Schizophyllum commune* [54] and *Chroogomphus rutilus* [55], showing that these two mushroom species could produce extracellular melanins with physical and chemical properties similar to intracellular melanins extracted from other fungi. The extracellular melanin from *S. commune* also exhibited the pronounced bioactivities including antibacterial and free-radical scavenging activities [54], meriting further development of scale-up production.

The draft genome of *Amorphotheca resinae* KUC30009, a fungal strain with promising industrial-scale melanin production capacity, was recently reported [56]. Its genome size was 30.11 Mb, containing 9638 predicted genes. Fourteen biosynthetic gene clusters (BGCs) associated with secondary metabolite productions were identified using genomics-based discovery analyses. Intriguingly, the genes encoding a specific type 1 polyketide synthase and 4-hydroxynaphthalene reductase were characterized to produce intermediate metabolites of the dihydroxy naphthalene (DHN)–melanin biosynthesis pathway, but they do not proceed to synthesize DHN–melanin. Additionally, the formation of melanin in the culture filtrate was observed to depend on the laccase-like activity of multi-copper oxidases, and melanin production was found to be proportional to laccase-like activity [56]. Thus, the above-described study may contribute to develop novel strategies for the improvement of melanin yields in culture filtrates.

Along with the quest for the fungal producers of extracellular melanin, the demands for novel melanized fungal species are surging, particularly for those dwelling in untapped niches and extreme environments that harbor extremophilic fungi.

Tudor et al. (2012) tested several *Ascomycetes* and *Basidiomycetes*, among which *Xylaria polymorpha* (an ascomycete) has been widely recognized to produce melanin via a polyketide pathway and *Trametes versicolor* (a basidiomycete) is known to biosynthesize melanin via the catechol pathway [57]. The melanin deposition in these species with characteristic spatial demarcation was found to be triggered by water limitation. Desiccation induces fungal species such as *X. polymorpha* to develop an adaptative strategy to ensure their survival. These dark-zone-line fungi were observed to yield melanin pigment that surrounds the fungal community, blocking water exchange within the wood substrate. Their studies investigated the effects of moisture content of specific wood substrates on fungal pigmentation, thereby contributing to effective manipulation of fungal pigmentation for spalting production. The ability to manipulate fungal pigmentation by altering moisture content in wood offers an opportunity to enrich the coloration intensity and inlay patterns associated with spalted woods. In addition, it also confers the high value-added features to untapped and/or underexplored hardwood species.

Accumulating evidence has revealed cryospheric fungi as an important source of isolating pigmented species and bioprospecting. Fungi isolated from the cryosphere have been found to produce melanin pigments with multifaceted applications [58,59]. For example, black yeasts are able to produce melanin and thick cell walls that protect them from solar radiation and other environmental stresses. Selbmann et al. [60] described the stunning tolerance of *Cryomyces antarcticus*, a rock-inhabiting fungus isolated from the continent of Antarctica, against UV irradiation while *Saccharomyces* (as the experimental control) was killed by a fraction of the UV dose that left the black fungus undisturbed. In addition, endophytic fungi recovered from the leaves of *Deschampsia antarctica* (Antarctic grass) were reported to be major producers of melanin, including black molds (about 80%) [61].

More recently, Pacelli et al. (2020) investigated the black fungus *Cryomyces antarcticus* via multidiscipline approaches, demonstrating that (1) pathways for producing DOPA and DHN are both active even in its extreme habitat, with the first one being more abundant than the second, and (2) melanin pigments are important for radioprotection, as manifested by the presence of melanin mixture for enhanced radio-resistance in this fungus [62]. Future research is needed for elucidating the precise relationship between the two melanin synthesis pathways and how the presence of two different types of melanins fortified radioprotection. More extensive understanding about the structure–activity functionality relationships is indispensable for the development of melanin-based biotechnology and bioengineering.

Psychrophilic fungi along with their secondary metabolites such as melanin and other types of colorants are increasingly serving as sustainable and valuable resources for biotechnological applications. Cryospheric fungi may be explored as the sources of colorants that are barely available to the industry, as exemplified by the characterization of blue-pigment-producing *Antarctomyces pellizariae* sp. nov. recovered from Antarctic snow [63].

Undoubtedly, agricultural and industrial practices generate a plethora of byproducts and wastes such as straw, stalk, husk, peel, seeds, bagasse, and plastics. Fermentation can use these underutilized and low-value agro-industrial residues and byproducts as substrates to generate high-value-added products including fungal colorants. Furthermore, the utilization of agro-industrial wastes as feedstocks for implementing the fungal transformation of valuable secondary metabolites would open the avenue for ecofriendly, sustainable, and cost-effective production of these colorants.

Despite a few reports pertaining to the usage of agro-industrial wastes for colorant production including carotenoids, Gamze (2021) was the first to describe using agro-industrial waste residues as fermentation substrate for fungal melanin production. The highest intracellular (0.19 g/L) and extracellular (3.52 g/L) melanin levels were yielded by *Aureobasidium pullulans* NBRC 100716 using carrot peel extracts as fermentation media [64]. Results showed that (1) carrot peel was a good feedstock for the large-scale production of high value-added melanin, and (2) characteristics of the melanin produced by *Au. pullulans* NBRC 100716, upon supplementation of carrot peel into fermentation medium, were similar to those of chemically synthesized melanin.

Due to recent advances in genetic and metabolic engineering, as well as other fungal cultivation innovations such as fermentation optimization and circularly harnessed feedstock, large-scale production of fungal melanin has improved significantly, with increasingly broad applications [65].

Melanin can be a desired alternative for the bioremediation of contaminated environments caused by heavy metals [66,67,68]. Melanin’s negative charge and large surface area allow the formation of ionic complexes with various metal ions under different conditions [69]. For instance, the utilization of melanin as a biosorbent has been exploited in the bioremediation of polluted waters. This strategy stems from the fact that, in addition to its metal-binding properties, melanin is tolerant to extreme temperatures, as well as insoluble in both aqueous solutions and organic solvents, thereby ensuring the stability of melanin-based biosorbents to curb heavy-metal-contaminated waterways, as well as the recovery of metals ions of high added-value from solutions [68]. Previous work revealed that melanin could serve as an efficient biosorbent to remove uranium from the aqueous system [66,67]. Melanin displayed good uptake over a wide pH spectrum, and absorption of uranium was observed to be rapid, the equilibrium of which reached within 2 h of treatment [66]. Tran-Ly et al. (2020) developed an electrospinning technique which incorporates fungal melanin extracted from *Armillaria cepistipes* (Empa 655), a novel source of adsorptive entities, into polymeric nanofibrous membranes to achieve highly porous filtration systems. At the toxic levels of Pb^2+^, Cd^2+^, Ni^2+^, and Cr^3+^, melanized membranes were found to be able to remove more than 90% of heavy metals in aqueous solutions, similar to those by raw melanin [70], suggesting that melanin derived from the mushroom *Armillaria cepistipes* is a promising biosorbent for the detoxification of heavy-metal ions from polluted waterways.

Superior affinity to metals by fungal melanins has also found application in medical settings, as manifested by oral supplements of iron–melanin for treating Fe-deficiency anemia. Song et al. (2016) conducted an investigation on the bioactivity of a *Lachnum* YM226 melanin–iron complex in the rodent model, demonstrating that this fungal–melanin–iron complex could considerably improve the decreased hemoglobin (Hb) level, and normalize the serum iron (SI) level, total iron-binding capacity (TIBC), and serum ferritin (SF) of the anemic mice in a dose-dependent manner [71].

Over the last few years, melanin nanoparticles have attracted great interest from academia, theranostics, and industry. Radiotherapy methodology such as external beam radiation therapy may cause detrimental effects to the bone marrow of cancer patients undergoing the therapy. Melanin-covered nanoparticles prepared by enzymatic polymerization on silica have been demonstrated to confer protection to bone marrow against radiotoxicity, as reflected by higher white blood cell and platelet counts post radiation therapy compared to the untreated control, allowing the administration of substantially higher doses targeting tumors [72]. In addition, the melanin-coated nanoparticles did not interfere with tumor treatment [72].

With the soaring interests toward nanomaterials that are biocompatible with a variety of tissues and organisms, several technologies and methodologies have been developed to fabricate melanin-based nanoparticles, including polydopamine-derived multifunctional coatings that are bioinspired by natural melanin biosynthesis. For instance, polydopamine (PDA) nanoparticles (sized less than 100 nm) have shown good biocompatibility with HeLa cells after proper surface modification, as well as free-radical-scavenging activities [73]. Another study revealed melanin-based nanoparticles as an ideal drug carrier in pH-responsive formulations for colon- and intestine-targeted pharmaceutical delivery [74].

Aside from antitumor applications, melanin has been registered for its potent anti-stress properties. In addition, Chyizhanska and Beregova proposed that fungal melanin produced by the black yeast *Exophiala nigra* X-1 (previously *Nadsoniella nigra*) could be of importance in the livestock industry. Their study with pigs showed that porcine weaning-associated morbidity and mortality were significantly abrogated after the administration of melanin to piglets (0.1 mg/kg) at weaning, a stressful period of time for most piglets [75].

## 4. Fungal Polyketide-Derived Colorants

Fungi are capable of biosynthesizing a diversity of colorful polyketide compounds, such as *Monascus* pigments of an azaphilone nature, the production of which remains undiscovered in other organisms such as higher plants [76]. Anthraquinones, naphthoquinones, and azaphilones are the representative classes of polyketide-derived colorants produced by fungi. The biosynthesis-associated genes for polyketide colorants in representative fungi are shown in Table 3.

Fungal anthraquinones are of various color shades, which give a yellow, orange, or brown hue to the mycelium of microscopic fungi, as well as to the fruiting bodies of macroscopic fungi (mushroom) [6]. In addition to their widely recognized roles in multifaceted industrial sectors such as food, textile, cosmetic, and drug discovery, more innovative applications are being described for fungal anthraquinones. For instance, in the field of microelectronics, anthraquinones were reported to act as organic semiconductors [80]. The semiconducting property of the above-described anthraquinone colorants produced by an endophytic fungus from *Lagerstroemia speciosa* (Queen flower) may be attributed to its structural characteristics of being hydrogen donors and acceptors, which makes them suitable as an intriguing charge transfer complex. Moreover, another investigation revealed that anthraquinone under redox reaction displayed significant switching characteristics [81], suggesting its potential utilization in molecular electronic devices, with potential advantages over the current silicon-based semiconductor components.

As a blue-green quinonic colorant produced by *Chlorociboria,* xylindein’s utilization in the field of (opto)electronics represents a novel and cutting-edge technology of fungal colorants. The excellent physical properties of xylindein, exhibiting a π-conjugated core structure and aggregate formation, enable electron transport with mobility of up to −0.4 cm^2^/(V·s) in amorphous xylindein films and potentially higher in high-purity xylindein [82,83,84]. Accordingly, xylindein is a potential candidate for sustainable organic electronics, thereby contributing to environmentally friendly technologies and the circular economy.

Recent studies based on bioactivity-guided designs generated several research outcomes with respect to screening and optimization of naphthoquinone production. Because naphthoquinones have allelochemical activities, cocultivation of the fungus of interest with another organism is expected to trigger naphthoquinone production [85]. For instance, exposure of *Fusarium fujikuroi* to ralsolamycin, derived from the bacterium *Ralstonia solanacearum*, was observed to induce the production of bikaverin, an antimicrobial naphthoquinone colorant in *Fusarium fujikuroi* [86]. Another investigation (2019) showed that cocultivation of the endophytic fungus *Fusarium tricinctum* with *Streptomyces lividans* led to the production of four new naphthoquinone dimers, namely, fusatricinones A–D [87]. More recently, Li et al. (2020) reported the identification of 1,4-naphthoquinone co-products, designated as monasones, in *Monascus ruber* M7 and unveiled their antimicrobial activities. Their work also found that monasones of *M. ruber* M7 could be stimulated by cocultivation with other challenging microorganisms. Altogether, these results suggested that the biosynthesis of naphthoquinone monasones in *M. ruber* provides an ecological benefit that helps this fungus defend its niche. This is consistent with the proposition by Medentsev and Akimenko (1998) that fungal naphthoquinone compounds play an important role in interactions with other microbes, plants, and animals [88].

*Monascus* species are able to synthesize a mixture of azaphilone pigments of varying constituents, amongst which six are well known as *Monascus* pigments (Mps) [89]. Mps have been used in food as colorants and additives for centuries in the Oriental regions, highly credited by the food industry in eastern Asian countries such as Korea, Japan, and China.

**Figure 2:**
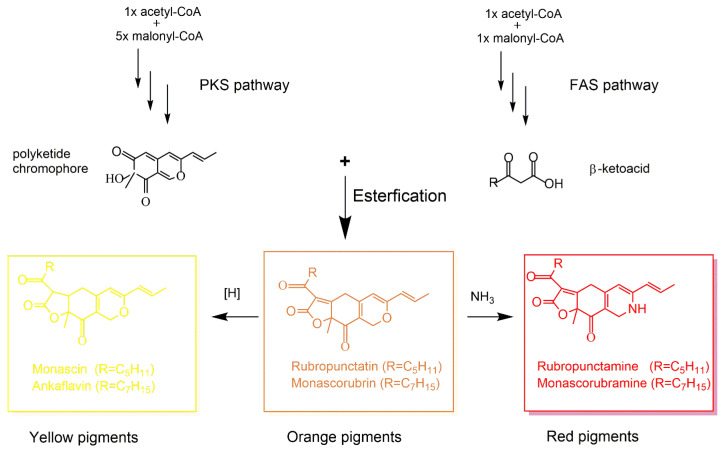
The schematic representation of pathways of six classical Mps biosynthesized by *Monascus* species.

The biosynthesis of Mps involves the polyketide pathway, where polyketide synthase (PKS) and fatty acid synthase (FAS) play crucial roles [90]. As shown in Figure 2, acetyl-CoA and malonyl-CoA are converted into *β*-ketoacid via the FAS pathway. Next, *β*-ketoacid reacts with the polyketide chromophore generated by the PKS pathway, leading to the production of orange pigments [91], which are further reduced to the yellow pigments via the hydrogenation reaction. Moreover, the orange pigments may be readily converted into red pigments via spontaneous amination by reacting with ammonia [92].

However, *Monascus* spp. can coproduce citrinin. Citrinin is a mycotoxin with nephrotoxicity and hepatotoxicity in mammals, and it is likely carcinogenic. Therefore, simultaneous production of citrinin in most *Monascus* species has limited the application of Mps in foods, as exemplified by the banned usage of MPs in the United States and European Union. For dozens of years, researchers have taken efforts to search for *Monascus* strains and species that are citrinin-free or only produce amounts of citrinin below the legislated level [93]. Although MPs and mycotoxin citrinin are produced from the same tetraketide, their biosynthetic routes proceed independently of each other [94], suggesting that enzymes responsible for colorant and citrinin production likely have independent mechanisms regulating their respective biosynthetic genes.

Genetic manipulation has been extensively applied to attenuate citrinin production. A polyketide synthase (PKS) gene, designated as *pksCT*, was cloned in *M. purpureus* and characterized as the gene responsible for citrinin biosynthesis, as evidenced by a high correlation between *pksCT* transcription and citrinin production [95]. Moreover, a *pksCT*-disrupted strain of *M. purpureus* abolished the production of citrinin, and a *pksCT* revertant, generated by successive endogenous recombinant events in the *pksCT* mutant, restored citrinin production. In accordance with this finding, deletion of *pksCT* in *Monascus aurantiacus* was subsequently reported to lead to a remarkable decline in citrinin production. Strikingly, the production capacity of red and yellow *Monascus* pigments was still maintained in this mutant [96]. Of note, biosynthesis pathways of fungal secondary metabolites including Mps and citrinin are known to be tightly regulated at the transcriptional level. The ensuing studies by Shimizu et al. identified a major transcriptional activator *CtnA* during citrinin biosynthesis in *M. purpureus*. Deletion of *ctnA* dramatically lessened the level of the *pksCT* transcript, resulting in reduced citrinin content [97].

In addition to the increasing genetic knowledge on citrinin production, further investigations on the regulatory mechanisms of colorant biosynthesis and the optimization of culture and large-scale fermentation are needed. It is necessary to maintain the yields of high-valued Mps during the establishment and development of citrinin-free or low-citrinin *Monascus* strains for Mps manufacture that fulfill the legislated threshold of food colorants/additives recognized internationally.

Application of a low-frequency magnetic field (LF-MF) has been reported to decrease citrinin production by *M. purpureus* in shake-flask liquid culture [98]. Under 30 °C, six different magnetic field induction intensities (MF-II), four different exposure times, and five exposure periods were examined to determine optimal treatment conditions. The cultures were exposed to a MF-II of 1.6 mT from 0 to 2 days of incubation time. With LF-MF treatment, peak citrinin production was found to decrease by 46.7%, while the production of yellow, red, and orange colorants and monacolin K increased by 31.3%, 40.3%, 41.7%, and 29.3%, respectively, as compared to the untreated controls at 12 days post incubation. Moreover, the relative expression levels of the citrinin biosynthesis genes *pksCT* and *ctnA* were 0.46 and 0.43 times lower, respectively, than the control. This study pinpoints LF-MF as a favorable means of altering *M. purpureus* metabolism to attenuate citrinin production and to promote Mps and monacolin K production without affecting cell growth. Accordingly, LF-MF may be a potential method to process *Monascus* products for beneficial food colorants/additives while minimizing the unwanted toxin citrinin.

The production of the citrinin by *Monascus purpureus* was also reported to be eliminated by adopting the fluconazole-treated medium [99]. Fluconazole is a known inhibitor of fungal ergosterol biosynthesis by acting on 14 *α*-demethylase. The lack of a characteristic band corresponding to the *R*_f_ value of standard citrinin on thin-layer chromatography (TLC) plates signaled colorant production but without citrinin in the fluconazole-supplemented fermentative medium. More recently, the changes in secondary metabolites of *Monascus aurantiacus* Li AS3.4384 (MALA) were reported using metabolomics analysis [100]. The metabolic profile changes were induced by genistein, an estrogen derivative, during fermentation. The supplementation of genistein was shown to reduce citrinin production due to the change in the precursor for citrinin biosynthesis in *Monascus*.

To select for a low-citrinin-content strain, *Monascus purpureus* YY-1, an industrial strain widely used in the manufacture of food colorants in China, was subjected to random mutagenesis using plasmid pHPH through protoplast transformation [101]. A random mutant designated as “winter” was obtained and studied for the biosynthesis and regulation mechanisms of colorant and citrinin in contrast to the parent strain *M. purpureus* YY-1. Comparative transcriptomic analysis demonstrated that *pksCT*, the essential gene for citrinin synthesis, exhibited a low expression level in *M. purpureus* YY-1 and the winter mutant. Moreover, Mps production by the winter mutant was elevated. Repressing the central carbon metabolism and enriching the acetyl-CoA pool contributed to a high Mps yield, and improved NADPH regeneration resulted in the metabolic flux of Mps production in *M. purpureus*. Investigations into the biosynthesis of citrinin and Mps, as well as the associated regulatory network in *M. purpureus*, will enhance our understanding of the metabolic and molecular bases underlying the biosynthesis of beneficial MPs and detrimental mycotoxin citrinin.

The effects of ammonium nitrate (NH_4_NO_3_) on the color features of Mps were recently investigated in *Monascus purpureus* M9 [79]. The concentration of intracellular colorants was significantly decreased in response to the ammonium nitrate supplement. The hue and lightness value indicated that ammonium nitrate could shift the overall appearance of colorants from originally red to orange. The HPLC analysis for six major components of Mps revealed that the yield of rubropunctatin or monascorubrin (orange colorants) was dramatically reduced to an undetectable level, whereas the productions of monascin and ankaflavin (yellow colorants), as well as rubropunctamine and monascorubramine (red colorants), were remarkably elevated by the supplementation of NH_4_NO_3_. It was found that, using real-time quantitative PCR, the expression level of *mppG*, highly associated with the biosynthesis of orange colorants, namely, rubropunctatin or monascorubrin, was significantly downregulated, while the expression of *mppE*, associated with the biosynthesis of yellow colorants, was upregulated. Moreover, the pH value of culture broth was dropped to 2.5–3.5 during the fermentation process, which was likely attributed to the addition of NH_4_NO_3_. The above-described findings are consistent with previous studies demonstrating that nitrogen significantly affects Mps biosynthesis [84,93]. Similarly, high salt stress could inhibit cell growth and hyphal development but upregulate colorant production. For example, elevated NaCl inhibited citrinin production and stimulated Mps production in *Monascus purpureus*, due to reduced expression levels of genes involved citrinin synthesis but enhanced expression of Mps genes [102].

Colorants are often produced by fungi as a biological manifestation in response to ecological and environmental stressors, such as light exposure, oxidizing agents, ionizing radiation, osmotic stresses, salinity, and temperature. However, fungal colorants are also synthesized to compete with and antagonize other organisms in the same ecological niches. An example of the second type is naphthoquinone, often produced by organisms in close proximity with others as a chemical defense [8].

To enhance our awareness about the environmental regulation of colorant quantity, an overview of the environmental factors impacting colorant production in fungi is given in Table 4.

## 5. Conclusions

The usage of colorants/additives constitutes an indispensable aspect of foods, cosmetics, and textiles for visual appeal and/or to compensate for and complement natural color variations. Despite increasing consumer demands and economic significance, the production of frequently used synthetic colorants is facing serious problems including heavy environmental burden, as well as the safety and health issues related to chemical processes and chemical effluents.

The food industry has a large demand for alternatives to synthetic food colorants. Fungi include a diversity of colorant-producing species and offer a variety of colorant molecules with the potentials of being developed as the sources of natural colorants. Fungal colorants have shown several advantages, including being highly stable under a diversity of light and temperature conditions, and being easier to cultivate and scale-up (in fermenters) than their plant counterparts. In addition, compared to plants, most fungal cell factories excel in their tractability to strain improvement, genetic modification, and metabolic engineering. A representative food-grade colorant example from *Xanthophyllomyces dendrorhous* is astaxanthin, a red-orange carotenoid dye with its use as a dietary additive in the aquaculture industry and potential in human health and nutrition [104]. In addition, many fungal colorants have shown excellent antioxidative, antiaging, photo-protective, antimicrobial, antitumor, and cholesterol-lowering activities [7], rendering fungal colorants valuable for the nutraceutical and cosmetic industry, or as lead molecules for medicine.

The wide-spectrum bioactivities and enormous potentials of colorants rationalize the bioprospecting of new sources of fungal colorants. The search for extremophilic pigmented fungi, e.g., in the cryosphere of the Antarctic, untapped marine habitats, and high irradiation environment, may bring intriguing discoveries. These are exemplified by the recoveries of novel blue-pigmented fungus *Antarctomyces pellizarize* (from Antarctic snow), three red-colored *Talaromyces albobiverticillius* strains producing *Monascus*-like pigments without citrinin (from the coral rubbles of the west coast of Reunion Island, Southwestern Indian Ocean), and hyper-melanized fungus *Paecilomyces lilacinus* (from the surrounding area of the Chernobyl nuclear plant) [58,105,106]. In-depth insight into and a full understanding of the ecological adaption and physiological plasticity of pigmented fungi in the harsh environments can facilitate the domestication and development of outstanding fungal strains using a combination of directed evolution, synthetic biology, genomics, transcriptomics, proteomics, metabolomics, etc. In addition, improvements are needed in order to increase the yields of desirable fungal colorants while minimizing the undesired byproducts and reducing costs. However, care should be taken in selecting species and strains. Not all colorful fungi are suitable hosts for producing natural fungal colorants at industrial scale for human use, even if they are easy to grow and can produce abundant colorants. Indeed, many plant, animal, and human pathogenic fungi such as *Alternaria alternata, Cryptococcus neoformans*, and *A. fumigatus* produce abundant melanin to help their survival inside and outside of their hosts [107] and such fungi won’t be ideal organisms for producing colorants at industrial scale. Together, concerted efforts from diverse approaches should lead us into a new era in which fungal colorants may completely replace synthetic colorants and serve as valuable lead molecules for novel medicines.

## Figures and Tables

**Table 1:** The genes involved in fungal biosynthesis of carotenoids.

Class of Carotenoids	Species	Biosynthesis-Related Genes	References
β-Carotene	*Blakeslea trispora*	*carRA, carB*, *carG*	[12,13]
	*Xanthophyllomyces dendrorhous*	*crtE*, *crtI*, *crtYB*	[11]
	*Rhodotorula glutinis*	*crtI*, *crtYB*	[14]
Astaxanthin	*Phaffia rhodozyma* (syn. *Xanthophyllomyces dendrorhous*)	*crtYB*, *crtS* (also called *Asy*), *crtI*, *crtE*	[15]
Lycopene	*Blakeslea trispora*	*carB*, *carRA*	[16]

**Table 2:** The genes involved in biosynthesis of fungal melanins.

Type of Melanin Biosynthesis Pathway	Species	Biosynthesis -Related Genes	References
DHN–melanin	*Aspergillus fumigatus*	*pksP/alb1, ayg1, arp1, arp2, abr1, abr2*	[47]
	*Aspergillus niger*	*alb1, ayg1, abr1, abr2*	[49]
	*Aspergillus nidulans*	*ωA, yA*	[50]
	*Penicillium marneffei*	*alb1, ayg1, arp1, arp2, abr1, abr2*	[49]
DOPA–melanin	*Cryptococcus neoformans*	*Lac1, Lac2*	[51]
Pyomelanin	*Aspergillus fumigatus*	*HppD, HmgA*	[45]
	*Penicillium chrysogenum*	*HppD, HmgA*	[46]

**Table 3:** Genes involved in the biosynthesis of polyketide colorants in represented fungi.

Class of Colorants/Paradigm	Fungal Species	Biosynthesis-Related Genes	References
Anthraquinones/endocrocin	*Aspergillus fumigatus*	*encA*, *encB*, *encC*	[77]
Naphthoquinone/bikaverin	*Fusarium fujikuroi*	*Bik1, Bik2, Bik3, Bik4, Bik5, Bik6*	[78]
Azaphilone/*Monascus* pigments	*Monascus purpureus*	*MppD*, *MpPKS5*, *MpFasB2*, *MpFasA2*, *MppF*, *MppA*, *MppB*, *MppE*, *Mpp7*, *MppC*, *MppG*, *MppR1*, *MppR2*	[79]

**Table 4:** Environmental factors that impact colorant production in fungi.

Categories of Colorants	Environmental Factors	References
Carotenoids	Light, temperature, oxygen, carbon source (i.e., glucose, ethanol), nitrogen, Ca^++^ availability	[24]
Melanins	UV irradiation, cold, desiccation, heavy-metal stress	[58,59]
Polyketide-derived pigments	Biotic stresses (i.e., other organisms), low-frequency magnetic field, NaCl, pH, NH_4_NO_3_	[79,102,103]

## Data Availability

This is a review article. No new original data were presented in this manuscript.
